# Rapidly evolving therapeutic advances for classical *EGFR*-mutant NSCLC

**DOI:** 10.3389/fonc.2025.1732467

**Published:** 2025-12-08

**Authors:** Kelsey Pan, Suresh S. Ramalingam

**Affiliations:** Department of Hematology and Medical Oncology, Emory University Winship Cancer Institute, Atlanta, GA, United States

**Keywords:** EGFR, NSCLC, targeted therapy, osimertinib, ADC

## Abstract

Epidermal growth factor receptor (*EGFR*)-mutant non-small cell lung cancer (NSCLC) has exemplified the advancement of precision oncology, yet inevitable resistance to tyrosine kinase inhibitors (TKIs) remains a challenge. Over the past decade, *EGFR* targeted therapies have extended beyond metastatic disease into early-stage and locally advanced settings, as demonstrated by ADAURA, NeoADAURA, and LAURA studies, which established osimertinib as the standard of care therapy across disease stages. Despite these advances, questions remain regarding the role of chemotherapy, duration of adjuvant targeted therapy, and the integration of ctDNA-guided minimal residual disease (MRD) monitoring in the early-stage setting. For metastatic disease, frontline combination strategies, such as osimertinib plus chemotherapy (FLAURA2) and amivantamab plus lazertinib (MARIPOSA), building on the *EGFR*-TKI backbone have improved progression-free and overall survival, particularly in higher-risk subgroups. However, as more therapeutic options emerge in the frontline and beyond, optimal treatment selection and sequencing become increasingly complex and tailored to individual risk factors, patient preferences, and disease biology. Following progression on third-generation TKIs, potential avenues for overcoming resistance include mechanism-based strategies targeting MET amplification or *EGFR* C797S, as well as mechanism-agnostic approaches such as bispecific antibodies and antibody-drug conjugates (ADCs). Collectively, these recent advances reflect the dynamic nature of the therapeutic landscape for *EGFR*-mutant NSCLC, which is becoming increasingly individualized, mechanism-informed, and resistance-adaptive, in efforts to achieve durable systemic and intracranial disease control.

## 1 Introduction

Epidermal growth factor receptor (*EGFR*) mutations represent one of the most common actionable oncogenic drivers in non–small cell lung cancer (NSCLC), affecting approximately 10-15% of patients with advanced disease in Western countries and up to 40–50% in Asia (1). The “classical” *EGFR* alterations, exon 19 deletions and L858R point mutations, are highly sensitive to *EGFR* tyrosine kinase inhibitors (TKIs) and have historically modeled precision oncology in thoracic malignancies. In contrast, atypical *EGFR* variants, such as exon 20 insertions and less common point mutations (G719X, S768I, L861Q), often exhibit primary resistance to currently available TKIs, underscoring the biological heterogeneity of *EGFR*-driven lung cancer (2). Despite the success of third-generation TKIs such as osimertinib, major unmet needs remain, including the treatment of on- and off-target resistance mechanisms and achieving durable central nervous system (CNS) and leptomeningeal disease (LMD) control (3–5). The expanding frontline therapeutic options, including TKI monotherapy, chemo-TKI, and bispecific antibody-TKI regimens, have necessitated consideration of optimal sequencing, ideally based on biomarker-informed strategies.

Over the past few years, the treatment paradigm for *EGFR*-mutant NSCLC has expanded substantially across the disease continuum. Pivotal studies such as ADAURA, NeoADAURA, and LAURA have extended targeted therapy beyond metastatic disease into early stage and locally advanced settings, with implications for integrating minimal residual disease (MRD) testing to guide treatment duration (6–8). In parallel, novel combinations including chemo–TKI combinations, bispecific antibodies, antibody–drug conjugates (ADCs), are increasingly being tailored to individual risk factors, patient preferences, and resistance mechanisms in the metastatic setting (9, 10). With growing emphasis on developing CNS-active regimens and ctDNA-guided adaptive strategies, the treatment landscape for *EGFR*-mutant NSCLC has become increasingly personalized (11). This review will summarize recent advances, investigational strategies, and future directions addressing overcoming resistance, enhancing CNS control, and optimizing sequencing frameworks in classical *EGFR*-mutant NSCLC.

## 2 Perioperative *EGFR* targeted therapy

### 2.1 Adjuvant therapy

The integration of *EGFR* TKIs into the adjuvant setting for NSCLC with classical *EGFR* mutations was established by the landmark ADAURA study. The initial results revealed a striking benefit in disease-free survival (DFS), with a hazard ratio of 0.23 (95% CI, 0.18-0.30) in stage II-IIIA disease, and 82% reduction in the risk of CNS recurrences or death (6, 12). Extended follow-up demonstrated a significant overall survival (OS) benefit with osimertinib therapy (**Table 1**) (13). These unprecedented results not only led to the swift adoption of adjuvant osimertinib into clinical practice but also provided a framework for exploring adjuvant targeted therapy in other actionable alterations (14).

**Table 1:** Active phase II-III perioperative trials in early-stage resectable EGFR-mutant NSCLC.

Trial	Phase	Population	Intervention	Comparator	Primary endpoint(s)	Efficacy outcomes
Adjuvant Therapies						
ADAURA	III	Resected stage IB–IIIA	Adjuvant osimertinib x 3 yrs after resection ± chemo	Placebo	DFS, OS	4-year DFS: 73% vs. 38% (HR 0.27; 95% CI, 0.21-0.34) 5-year OS: 88% vs. 78% (HR 0.49; 95% CI, 0.34-0.70)
ADAURA2 (NCT05120349)	III	Resected stage IA2–IA3	Adjuvant osimertinib x 3 yrs after resection	Placebo	DFS	Ongoing
TARGET (NCT05526755)	II (single-arm)	Resected stage II–IIIB	Adjuvant osimertinib x 5 yrs	N/A	5-year DFS	Ongoing
Neoadjuvant ± Adjuvant Therapies						
NeoADAURA (NCT0435155)	III	Resectable stage II–IIIB	Neoadjuvant osimertinib ± platinum-pemetrexed x 3C → surgery → optional adjuvant osimertinib x 3 yrs ± adjuvant chemotherapy	Neoadjuvant platinum-doublet chemotherapy	MPR, EFS	Osimertinib + chemo vs. osimertinib monotherapy vs. chemo: MPR: 26% vs. 25% vs. 2% 12-mo EFS: 93%, 95%, 83% N2 nodal downstaging: 53% vs. 53% vs. 21%
EMERGING CTONG 1103 (NCT01407822)	II	Resectable stage IIIA-N2	Neoadjuvant erlotinib x 6 weeks → surgery → adjuvant erlotinib x 1 yr	Neoadjuvant gemcitabine/cisplatin x 2C → surgery → adjuvant gem/cis x 2C	ORR, PFS, OS	PFS: 21.5 vs. 11.4 mo (HR 0.36; 95% CI, 0.21-0.61) OS: 42.2 vs. 36.9 mo (HR 0.83; 95% CI, 0.47-1.47)
NCT05469022	II (single-arm)	Resectable stage I-IVA	Neoadjuvant lazertinib x 9 weeks → surgery → V adjuvant lazertinib x 3 years (for stage ≥II)	N/A	ORR, MPR, DFS	Ongoing
NCT0501187	II (single-arm)	Resectable stage III (N2)	Neoadjuvant osimertinib x 60 days + platinum-pemetrexed x 2C	N/A	Complete lymph node clearance rate, MPR, DFS	Ongoing
FORESEE (NCT0543082)	II (single-arm)	Resectable stage IIIA-IIIB	Neoadjuvant furmonertinib x 9 weeks + platinum-pemetrexed x 3C	N/A	ORR, MPR, DFS, OS	Ongoing
ANSWER (NCT04455594)	II	Resectable stage IIIA-N2	Neoadjuvant almonertinib	Erlotinib or platinum-pemetrexed x 3C	ORR, MPR, DFS, OS	Ongoing

Despite these remarkable results, several questions remain, including the utility of adjuvant chemotherapy, optimal duration of adjuvant targeted therapy, and the integration of ctDNA to guide clinical management. With regards to the role of adjuvant chemotherapy, ADAURA subgroup analyses showed consistent DFS and OS benefits whether patients received adjuvant chemotherapy or not, though the trial was not randomized to answer this question. Our opinion is that adjuvant cisplatin-based chemotherapy has documented ability to cure a subset of NSCLC while the same cannot be conclusively stated with regards to the role of targeted therapy in early-stage disease. Therefore, administration of chemotherapy in eligible patients as the first step prior to the initiation of osimertinib should be considered the standard of care for patients with early-stage *EGFR* mutant lung cancer after surgical resection (15).

The ADAURA study utilized osimertinib for a maximum of 3 years; unfortunately, a high proportion of patients develop disease recurrence after the discontinuation of targeted therapy. To address this issue, the TARGET trial is actively investigating whether extending osimertinib therapy for 5 years offers additional survival benefits compared to 3 or 4 years (16); the ADAURA2 is evaluating if adjuvant osimertinib will benefit patients with stage IA2–3 disease, since this subset was not included in the ADAURA trial (17). Recently, a *post-hoc* study of the ADAURA cohort showed that a tumor informed, circulating tumor DNA (ctDNA)-based MRD assay detected molecular relapse 4.7 months prior to radiographic DFS events, with most occurring after the 3-year duration of adjuvant osimertinib (18). Notably, 58% of all recurrences and MRD events occurred within 12 months of stopping osimertinib, suggesting that patients with detectable ctDNA may potentially benefit from a longer duration of adjuvant treatment. Hence, ongoing efforts are evaluating the role of dynamic monitoring of postsurgical ctDNA to identify those who may benefit from extended therapy duration (18–20).

### 2.2 Neoadjuvant and perioperative trials

Following the success of ADAURA, administration of osimertinib in the neoadjuvant setting was assessed by the phase III NeoADAURA trial. In this study, patients with stage II-IIIB resectable *EGFR*-mutant NSCLC were randomized to receive neoadjuvant osimertinib with or without chemotherapy versus chemotherapy alone prior to surgical resection; following surgery, systemic therapy-eligible patients (over 90%) were offered adjuvant osimertinib. Early results with median follow up of approximately 14 months suggest that neo-adjuvant osimertinib demonstrated significant improvement in major pathologic response (MPR) rates (approximately 25% vs. 2%, respectively), and nodal downstaging in 53% of patients with N2 disease, compared to 21% in those receiving chemotherapy alone (**Table 1**) (7). Notably, the MPR and early EFS outcomes were comparable between the group treated with neoadjuvant osimertinib alone versus osimertinib plus chemotherapy. However, it is premature to conclude that osimertinib alone is sufficient given that the benefit of adding chemotherapy may not manifest in the short-term.

It is important to note that the prognostic value of MPR [especially in absence of pathologic complete response (pCR)] in relation to long-term survival has not yet been established for targeted therapies. Less than 10% of the patients achieved pCR with osimertinib given alone or with chemotherapy, which is disappointing. Since over 90% of patients in both groups received adjuvant osimertinib, it is unlikely that a difference in overall survival will be noted even with longer follow up (21). Regardless, these results support the use of pre-operative osimertinib with platinum-based chemotherapy in patients with N2 disease prior to surgery, given that upfront surgery is not recommended for this subset. The planned studies on MRD to correlate with EFS and OS are eagerly awaited to determine whether therapy could be individualized to patients.

### 2.3 Unresectable stage III

For patients with unresectable stage III disease, the post-chemotherapy strategy remained undefined until the LAURA trial, as those with *EGFR* mutations derived limited benefit from consolidation durvalumab (8, 22). LAURA instantly established consolidation osimertinib following chemoradiation as the new standard-of-care for this population, based on the impressive progression-free-survival (PFS) benefit of 39.1 months with osimertinib versus 5.6 months with placebo (HR 0.16, 95% CI, 0.10-0.24). Importantly, osimertinib significantly reduced the incidence of new CNS metastases, local and distant metastases. However, given that osimertinib was continued indefinitely until disease progression in this trial, ctDNA-based risk stratification has emerged as a crucial area of investigation in this setting. Post-chemoradiation ctDNA positivity has been consistently associated with increased risk of recurrence and inferior outcomes, suggesting its potential role in guiding de-escalation strategies and optimizing treatment duration (23, 24). The final analyses of OS, CNS-specific PFS, and ctDNA-based exploratory analyses of the LAURA study will be essential in delineating the long-term outcomes of this approach. The POLESTAR study which evaluated aumolertinib, another 3^rd^ generation TKI in locally advanced NSCLC, also noted efficacy results comparable to the LAURA trial, thus confirming the efficacy of *EGFR* inhibition in this setting (25).

## 3 Metastatic NSCLC: front line therapy

### 3.1 TKI monotherapy backbone and chemo-TKI combinations

Third-generation *EGFR* TKIs (osimertinib, lazertinib, aumolertinib, furmonertinib, etc.), provide durable systemic disease control and CNS penetrance, and have become the cornerstone of frontline therapy for metastatic NSCLC with classical *EGFR* mutations. The FLAURA trial established osimertinib as the standard of care by demonstrating both improved progression-free survival (median 18.9 vs. 10.2 months; HR 0.46; 95% CI, 0.37-0.57) and OS (median 38.6 vs. 31.8 months; HR 0.80; 95% CI, 0.64-1.00) compared to earlier-generation *EGFR* TKIs (26, 27). Importantly, it also showed improved CNS PFS, which was not previously achieved or routinely evaluated in studies of earlier-generation *EGFR* TKIs (27). Building on this TKI backbone, FLAURA2 evaluated the addition of platinum-pemetrexed chemotherapy to osimertinib as frontline treatment in this population. Compared to osimertinib monotherapy, the addition of chemotherapy yielded a notable improvement in progression-free survival (25.5 vs. 16.7 months; HR 0.62; 95% CI, 0.49-0.79), with an especially pronounced benefit among patients with baseline CNS metastases (24.9 vs. 13.8 months, HR 0.47; 95% CI, 0.33-0.66) and L858R mutations (28). Osimertinib plus chemotherapy also showed delayed CNS progression in patients with and without baseline CNS metastases, as well as improved CNS ORR (73% vs. 69%) and CNS PFS (HR 0.58; 95% CI, 0.33-1.01) among patients with baseline CNS metastases (29). In patients with baseline CNS metastases, the intracranial ORR These benefits also translated into a significant improvement in overall survival for the addition of chemotherapy to osimertinib (47.5 vs. 37.6 months, HR 0.77; 95% CI, 0.61-0.96) (30).

It is important to consider the trade-off of increased grade ≥3 adverse events (AEs) (57% vs 36%) and treatment discontinuation, mostly due to chemotherapy-related toxicities, such as anemia (20% grade 3), neutropenia (11%), thrombocytopenia (6%), diarrhea (3%), decreased appetite (3%) and fatigue (3%). Less quantifiable but equally important are the quality-of-life impacts that chemotherapy infusion schedules may add; thus, shared decision making with the patient remains a crucial component of treatment selection. Especially for patients with higher risk disease biology, such as L858R mutation, baseline CNS metastases, or high tumor burden, chemo-TKI may be favored, while osimertinib monotherapy remains a reasonable option for those with lower risk disease who may not be a candidate for chemotherapy. As more options enter the frontline treatment paradigm, identification of biomarkers to risk-stratify patients who may benefit from treatment intensification is critical.

### 3.2 Bispecific antibody–TKI strategy

Beyond TKI combinations with chemotherapy, a bispecific antibody approach has emerged as a strategy to enhance TKI efficacy in the frontline setting. Amivantamab, a bispecific antibody with dual-receptor blockade of *EGFR* and MET, was evaluated in combination with lazertinib (3^rd^ generation *EGFR* TKI) versus osimertinib monotherapy in the MARIPOSA trial (31). This combination approach, combining *EGFR* inhibition with targeting a common resistance mechanism (i.e. MET pathway), demonstrated both progression-free survival (23.7 vs. 16.6 months, HR 0.70; 95% CI, 0.58-0.85) and overall survival (NE vs. 36.7 months, HR 0.75; 0.61-0.92) benefits (32). It also demonstrated CNS PFS benefit in those with brain metastases, and a significant PFS benefit in high-risk subgroups, including those with TP53 co-mutations, detectable baseline ctDNA, and liver metastases (33).

Amivantamab comes with distinct toxicities that require proactive management; the most common grade ≥3 adverse events include rash (15%), paronychia (11%), dermatitis acneiform (8%), pulmonary embolism (5%), and infusion-related reactions (IRR) (6%); it is important to note that venous thromboembolism was reported in 37% of the patients receiving amivantamab-lazertinib (31). Strategies such as prophylactic medications and enhanced skin care to mitigate infusion reactions and dermatologic toxicities are being studied in ongoing clinical trials (34, 35). Subcutaneous amivantamab, which demonstrates noninferior pharmacokinetics and efficacy (by ORR) compared with IV amivantamab in PALOMA, is another strategy that could decrease risk of infusion reactions (though risk of other toxicities remain the same), in addition to improving administration logistics and patient convenience (36). Taken together, the combination of amivantamab and lazertinib is associated with both clinical benefit and considerable toxicity concerns that merit careful discussion with patients prior to treatment initiation.

As more innovative therapies and combinations enter the frontline space, sequencing after progression emerges as a growing area of uncertainty. As the bispecific antibody-TKI approach gets integrated into the treatment paradigm, mitigating toxicities, optimizing treatment delivery, and understanding post-progression sensitivity to TKIs and chemotherapy will be crucial, particularly in the context of FLAURA2 offering similar benefits overall and in high-risk subgroups.

## 4 Management after osimertinib progression

### 4.1 Resistance biology and testing at progression

**Figure 1:**
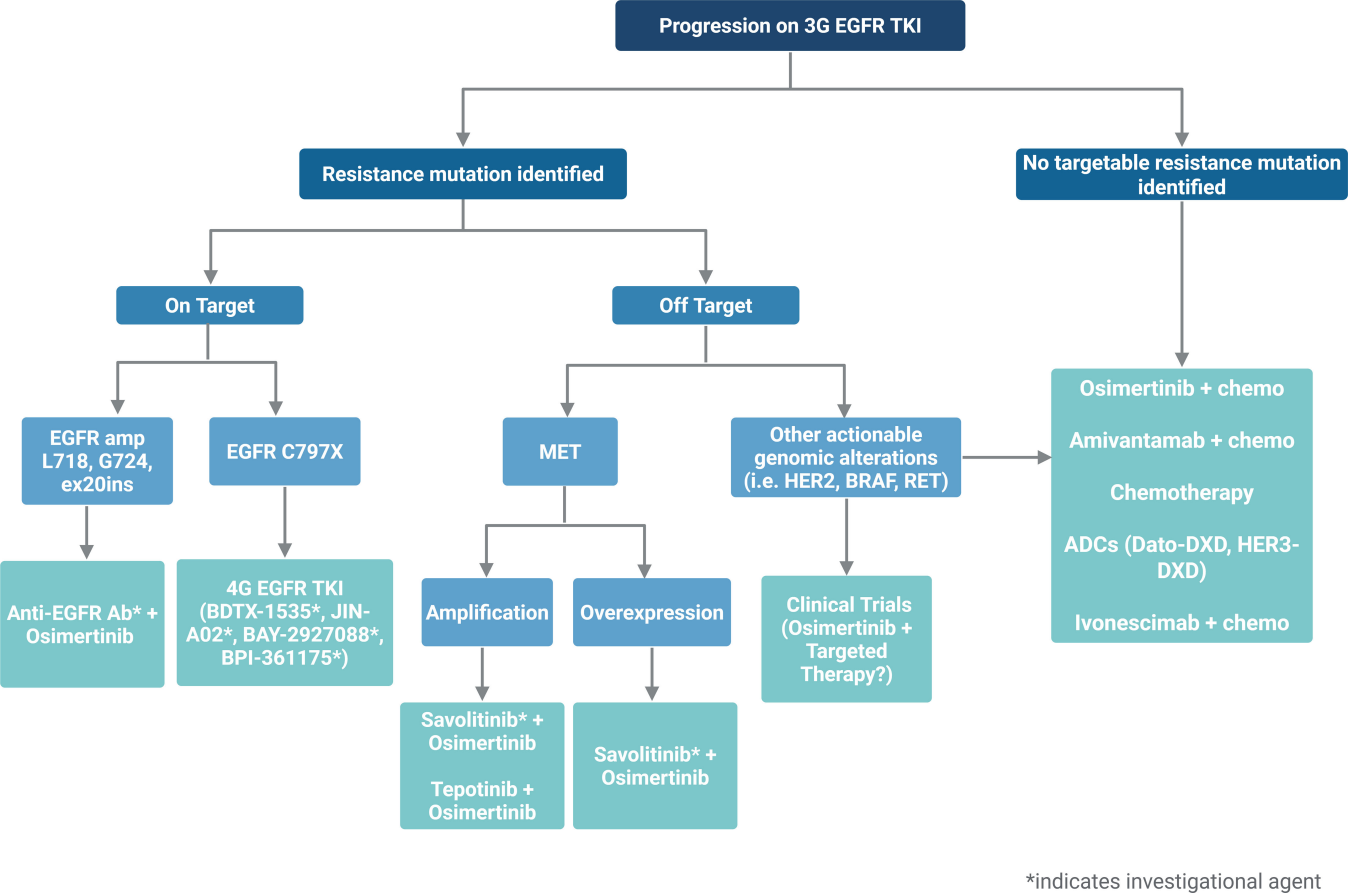
Potential treatment strategies based on mechanisms of resistance to third-generation EGFR tyrosine kinase inhibitors.

Eventual progression on third-generation *EGFR* TKI is inevitable, and understanding resistance mechanisms could guide subsequent-line therapy (**Table 2**). Resistance patterns are highly heterogenous, with on-target mechanisms such as *EGFR* C797S and exon 20 insertions, and off-target resistance ranging from bypass signaling through MET amplification/overexpression, HER2 activation, to histologic transformation into small-cell or squamous cell lung cancer (**Figure 1**) (4, 9, 37). Re-biopsy with molecular testing and/or plasma ctDNA testing are avenues to determine resistance mechanisms at the onset of acquired resistance; however, presently this information does not always inform subsequent therapy in routine clinical practice and hence is used primarily to identify potential clinical trials.

**Table 2:** Clinical trials evaluating subsequent-line therapies after progression on osimertinib in metastatic EGFR-mutant NSCLC.

Trial	Phase	Biomarker selection	Intervention	Comparator	ORR	PFS	OS	CNS PFS	Grade ≥3 AEs
Mechanism-agnostic approaches									
MARIPOSA-2	III	N/A	Amivantamab + Carboplatin + Pemetrexed	Carboplatin + Pemetrexed	64% vs. 36%	6.3 vs. 4.2 mo; HR 0.48 (0.36-0.64)	17.7 vs. 15.3 mo; HR 0.73 (0.54-0.99)	12.5 vs. 8.3 mo; HR 0.55 (0.38-0.79)	72% vs. 48%
COMPEL	III	N/A	Osimertinib + platinum-pemetrexed	Platinum-pemetrexed	35% vs. 29%	8.4 vs. 4.4 mo; HR 0.43 (0.27-0.70)	15.9 vs. 9.8 mo; HR 0.71 (0.42-1.23)	15.9 vs. 8.6 mo; HR 0.56 (0.27-1.13), in pts w/o baseline CNS mets	63% vs. 46%
HERTHENA-Lung02	III	N/A	Patritumab-deruxtecan	Platinum-pemetrexed	35.2% vs. 25.3%	5.8 vs. 5.4 mo; HR 0.77 (0.63-0.94)	16.0 vs. 15.9 mo; HR 0.98 (0.79-1.22)	5.4 vs. 4.2; HR 0.75 (0.53-1.06)	73% vs. 57%
TROPION-Lung05	II (single arm)	N/A	Datopotamab-deruxtecan	N/A	43.6%	5.8 mo	18.3 mo	N/A	28.5%
OptiTROP-Lung04 (China only)	III	N/A	Sacituzumab tirumotecan	Platinum-pemetrexed	60.6% vs. 43.1%	8.3 vs. 4.3 mo; HR 0.49 (0.39-0.62	NR vs. 17.4 mo; HR 0.60 (0.44-0.82)	N/A	58.0% vs. 53.8%
BL-B01D1 (China only)	I/II	N/A	lzontamab brengitecan	N/A	56.0%	12.5 mo	18-mo OS 69.2%	N/A	71% (in basket study)
HARMONi	III	N/A	Ivonescimab + platinum-pemetrexed	Platinum-pemetrexed	45% vs. 34%	6.8 vs. 4.4 mo; HR 0.52 (0.41-0.66)	16.8 vs. 14.0 mo; HR 0.79 (0.62-1.01)	N/A	50% vs. 42.4%
Mechanism-based approaches									
SAVANNAH	II (single arm)	MET overexpression/amp	Savolitinib + Osimertinib	N/A	56.3%	7.4 mo	N/A	N/A	51.0%
SACHI	III	MET amp	Savolitinib + Osimertinib	Platinum + pemetrexed	58% vs. 34%	6.9 vs. 3.0 mo; HR 0.32 (p<0.0001)	22.9 vs. 17.7 mo; HR 0.84 (p=0.42)	N/A	56.6% vs. 57.3%
INSIGHT-2	II (single arm)	MET amp	Tepotinib + Osimertinib	N/A	50.0%	5.6 mo	17.8 mo	7.8 mo	34%
ORCHARD	II (single arm)	EGFR amp, L718, G724, ex20ins	Necitumumab + Osimertinib	N/A	11%	4.0 mo	11.4 mo	N/A	53%

### 4.2 Mechanism-based subsequent therapeutic approaches

#### 4.2.1 MET-directed combinations for MET-driven resistance

MET amplification and overexpression represent the most common bypass mechanisms of acquired resistance to third-generation *EGFR* TKIs, with an estimated frequency of around 7-15% after first-line osimertinib and 5-50% after second-line osimertinib (38). The phase II SAVANNAH trial evaluated osimertinib plus savolitinib, a highly selective MET TKI, versus savolitinib monotherapy in patients with MET-amplified or overexpressed tumors following first-line osimertinib, as defined by MET IHC3+/≥90% (3+ staining intensity in ≥90% of tumor cells) and/or FISH10+ (≥10 MET gene copies) status (39). Using a novel biomarker-based strategy for patient selection, this trial established a framework for future studies designed to target biomarker-guided resistance mechanisms. The combination demonstrated an overall response of 56% and median progression-free survival of 7.4 months; the most common any-grade adverse events with the combination include peripheral edema (46.0%), nausea (40.5%), and diarrhea (23.2%), with the most common grade ≥3 AEs being peripheral edema (4.4%), pulmonary embolism (4.4%), pneumonia (4.1%), and dyspnea (4.1%).

Building on these findings, the phase III SACHI trial conducted in China compared savolitinib-osimertinib versus chemotherapy in patients with MET amplification, showing significantly improved progression-free survival (6.9 vs 3.0 months, HR 0.32; 95% CI, 0.23-0.49); the overall rates of grade ≥3 toxicities (56.6% vs. 57.3%, respectively) were comparable with lower rates of hematologic events in comparison to chemotherapy (40). In the savolitinib-osimertinib arm, the most common grade ≥3 adverse events included neutropenia (14%), leukopenia (7%), and increased alanine aminotransferase levels (7%), while in the group that received chemotherapy, neutropenia (26%) and anemia (23%) were the commonly observed grade ≥3 AEs. The phase III SAFFRON trial evaluating savolitinib-osimertinib versus chemotherapy in those with MET overexpression and/or amplification following osimertinib progression is currently ongoing to validate these findings in a global cohort.

Similarly, the phase II INSIGHT-2 study assessed tepotinib (MET inhibitor) with osimertinib in patients with MET amplification following osimertinib progression; the response rate was 50.0% and the mPFS was 5.6 months. The median overall survival was modest at 17.8 months (41). Altogether, these studies suggest a potential role for *EGFR*- and MET-inhibition as post-osimertinib therapeutic strategy in the MET-amplified patient subset; a notable gap in current knowledge is the lack of optimal definition for MET amplification/over expression.

#### 4.2.2 Secondary EGFR resistance mechanism-directed therapies

A small subset (<10%) of patients develop acquired resistance through on-target *EGFR* alterations, such as L718, G724, exon 20 insertions, and *EGFR* amplification. The phase II ORCHARD trial, a biomarker-directed platform study, evaluated osimertinib with necitumumab, an anti-*EGFR* monoclonal antibody, in patients with *EGFR* amplification, L718 or G724 mutation, or exon 20 insertion identified after progression on frontline osimertinib (42). The results including overall response rate of 11% (with 2 partial responses seen only in those with *EGFR*-amplified tumors), median progression-free survival of 4.0 months and overall survival of 11.4 months are of some interest, though unlikely to result in larger studies.

Resistance to osimertinib mediated by *EGFR* C797X (including C797S and C797G) mutation has been recognized as one of the most clinically relevant on-target *EGFR* mechanisms, as this mutation disrupts the binding pocket of third-generation TKIs to the kinase domain. *EGFR* C797X has been observed in approximately 8.0% of those treated with first-line osimertinib, and 17% of those treated with second-line osimertinib. While MET amplifications are more frequently observed during the first year of frontline osimertinib, *EGFR* C797X emerges as the most common mechanism of resistance following the first year (43). In attempt to overcome *EGFR* on-target resistance, fourth-generation *EGFR* TKIs have emerged as a therapeutic strategy. BDTX-1535 has demonstrated preliminary efficacy in patients with *EGFR*-mutant NSCLC who progressed on an *EGFR* TKI, with an overall response rate of 36% in the entire cohort and 42% in those with known *EGFR* C797S resistance mutation (44). Currently, several other fourth-generation oral *EGFR* TKIs, including JIN-A02, BAY 2927088, and BPI-361175, are under active investigation in early phase clinical trials (45). These agents represent a potential avenue in the post-osimertinib setting, and defining their optimal use—whether as monotherapy or in combination with other targeted or systemic therapies—will be important prior to clinical adoption.

### 4.3 Mechanism-agnostic therapeutic approaches

#### 4.3.1 Bispecific antibody + chemotherapy combination

The phase III MARIPOSA-2 trial evaluated the combination of amivantamab and platinum-pemetrexed chemotherapy with or without lazertinib in patients who progressed on frontline osimertinib (46). Both treatment arms receiving the amivantamab-chemotherapy combination demonstrated improvements in both systemic and intracranial progression-free survival compared to chemotherapy alone. However, these results have not translated into overall survival improvement at the time of the interim analysis. The addition of both amivantamab and lazertinib to chemotherapy did not appear to offer additional benefit beyond the triplet therapy of amivantamab-chemotherapy; hence, the FDA has approved chemotherapy with amivantamab for use in this setting.

As previously discussed, the high rate of associated toxicities with 72% grade ≥3 AEs, including 10% venous thromboembolism, and 65% dose interruptions with the triplet regimen are practical considerations in a decision to offer chemotherapy alone versus the triplet combination for patients.

#### 4.3.2 Chemotherapy with EGFR TKI backbone

Before the adoption of osimertinib combined with platinum-based chemotherapy as standard frontline treatment, a common strategy was to add chemotherapy at the time of progression on *EGFR* TKI monotherapy. The COMPEL study defined the efficacy of continuing osimertinib with chemotherapy versus treatment with chemotherapy alone in patients with non-CNS progression on frontline osimertinib monotherapy. Median progression-free survival nearly doubled in the osimertinib plus chemotherapy combination arm (8.4 months), compared to 4.4 months in the chemotherapy-only arm (HR 0.43; 95% CI, 0.27-0.70). Though not yet mature, there was a trend towards longer overall survival of 15.9 versus 9.8 months (HR 0.71; 95% CI, 0.42-1.23) and CNS PFS with combination therapy in those without baseline brain metastases (15.9 vs. 8.6 months, HR 0.56; 95% CI, 0.27-1.13) (47). As more subsequent-line novel therapies emerge, this study suggests a role for continuation of *EGFR* TKI backbone throughout subsequent-line treatments; this needs to be verified in an adequately powered clinical trial.

#### 4.3.3 Antibody-drug conjugates

ADCs are emerging as a promising class of therapeutics across a broad range of lung cancers, with the potential to overcome heterogenous resistance mechanisms of *EGFR*-mutant NSCLC after TKI progression. Patritumab deruxtecan is a HER3-directed ADC that has demonstrated meaningful clinical activity in those with progressive *EGFR*-mutant disease (48, 49). HER3 protein expression is seen in the vast majority of *EGFR*-mutant NSCLC, and translational studies demonstrate its upregulation following *EGFR* TKI progression, making it a potential therapeutic target in this setting (50). In HERTHENA-Lung02, patritumab demonstrated improved response rate (35% vs. 25%) and a statistically significant, yet clinically modest progression-free survival benefit (5.8 vs 5.4 months; HR 0.77, 95% CI, 0.63-0.94) compared to platinum-based chemotherapy. Similar rates of treatment-related AEs and treatment discontinuation were observed with both treatments, and patritumab showed a trend towards improved CNS PFS (5.4 vs. 4.2 months; HR 0.75, 95% CI, 0.53-1.06. However, the application for FDA approval was withdrawn based on the lack of overall survival benefit (16.0 months vs. 15.9 months). Ongoing early-phase trials explore patritumab in combination with osimertinib in effort to enhance efficacy.

Datopotamab deruxtecan (Dato-DXd) is a TROP2-directed ADC that has shown encouraging efficacy in treated *EGFR*-mutant NSCLC. In phase II TROPION-Lung05, Dato-DXd demonstrated a response rate of 44%, median progression-free survival of 5.8 months, and overall survival of 18.3 months in patients who previously received *EGFR*-TKI and platinum-based chemotherapy (51). Toxicity included 29% grade ≥3 TRAE, including stomatitis (9.5%), amylase increase (5.1%), anemia (2.9%), nausea (2.2%), and decreased appetite (2.2%), leading to treatment discontinuation in 5.1%. Combination strategies with osimertinib are also under investigation in the ORCHARD trial (52). Datoptamab deruxtecan has recently received FDA approval for patients with *EGFR* mutant lung cancer following progression on standard therapies. Another TROP2-directed ADC, sacituzumab tirumotecan, was recently shown to significantly prolong median progression-free survival (8.3 vs. 4.3 months, HR 0.49; 95% CI, 0.39-0.62) and reduce the risk of death by 40% (HR 0.60; 95% CI, 0.44-0.82) in this setting compared to chemotherapy in the phase III OptiTROP-Lung 04 trial (53). Currently, global phase 3 studies of sacituzumab tirumotecan alone and in combination with osimertinib are ongoing.

Additional emerging ADCs, such as the HER3-directed izalontamab brengitecan (54), are being actively investigated in early-phase clinical trials, further broadening therapeutic options. Furthermore, combinations of osimertinib with ADCs (such as Dato-DXD (40) and BL-B01D1) have demonstrated preliminary efficacy and continue to be actively investigated in ongoing clinical trials. As additional ADCs and other novel therapies enter the treatment landscape, incorporating biomarker assays, will be critical to predict response and identify patients most likely to benefit (55).

#### 4.3.4 Dual PD-1 and VEGF blockade

Beyond HER3 and TROP2 targeting, chemotherapy in combination with ivonescimab, a bispecific antibody targeting PD-1 and VEGF, has demonstrated efficacy in those progressing on a 3rd generation *EGFR* TKI. Immunotherapy has historically shown minimal to no efficacy in *EGFR*-mutant NSCLC, with multiple retrospective and prospective studies demonstrating low response rates to checkpoint inhibitors as monotherapy or in combination with chemotherapy (56–59). However, the phase III HARMONi trial, which evaluated ivonescimab in those who progressed on prior *EGFR* TKI, demonstrated improved progression-free survival of 6.8 months, compared to 4.4 months in those receiving chemotherapy alone, and a trend towards improved overall survival (60). While the progression-free survival benefit is only modest and comparable to studies evaluating VEGF inhibitors alone in this population, it is unclear whether the dual targeting of PD-1 and VEGF pathways truly overcomes the intrinsic resistance of *EGFR*-mutant tumors to traditional immunotherapy. The trial results were primarily driven by patients from China and therefore the efficacy in a broader patient population is yet to be fully characterized.

## 5 Local consolidation in oligometastatic disease

In select patients with oligometastatic disease progression, implementing local consolidative strategies such as surgical resection or ablative radiotherapy while continuing the *EGFR* TKI remains a common clinical strategy that is supported by retrospective and early prospective studies (61–64). More often applied in the oligoprogressive setting, a phase II trial assessed local ablative therapy followed by osimertinib rechallenge; there was a modest second PFS (PFS2) of 3.7 months (65). Although the primary endpoint was not met, the identification of a subgroup with lower tumor burden and negative baseline ctDNA/MRD who derived exceptional benefit highlights the potential utility of biomarker-guided patient selection for local ablative therapy.

For upfront treatment of oligometastatic disease, it remains uncertain whether attempting to eradicate sites of residual resistant clones through local consolidative therapy can truly delay systemic progression and translate into a benefit in overall survival. The phase II NORTHSTAR trial randomized patients to osimertinib with or without local consolidation therapy (LCT) as frontline therapy, demonstrating a benefit in progression-free survival (25.3 vs. 17.5 months; HR 0.66) among patients with both oligometastatic and polymetastatic disease (62, 66, 67). However, overall survival outcomes are still pending. Ongoing research aims to refine patient selection by distinguishing those with more favorable oligometastatic disease biology from those with more extensive micro-metastatic involvement, while defining how to sequence LCT with these rapidly evolving systemic treatments.

## 6 Future directions and conclusions

The therapeutic landscape of *EGFR*-mutant NSCLC continues to evolve rapidly, with several novel therapies, resistance targets, and synergistic combinations—including ADCs, bispecific antibodies, chemotherapy, and anti-angiogenic agents—under investigation to further refine optimal treatment sequencing. An emerging strategy is the integration of ctDNA to guide perioperative decision-making, such as adjuvant treatment intensification or adapting the duration of adjuvant treatment based on dynamic MRD monitoring. In the advanced/metastatic disease setting, combination therapy is becoming the new standard-of-care frontline treatment in select high-risk groups, such as those with high tumor burden, baseline CNS or liver metastases, TP53 co-mutations or detectable ctDNA, though careful patient selection and counseling are essential given the added toxicity.

After inevitable progression on *EGFR* TKIs, MET TKIs and next-generation *EGFR* inhibitors designed to overcome acquired on- and off-target resistance mechanisms, such as *EGFR* C797S, MET amplification and overexpression, represent rapidly growing areas of drug development. These agents are currently being investigated both as monotherapy and in rational combinations with 3^rd^ generation *EGFR* TKIs. Additionally, combinations incorporating chemotherapy, bispecific antibodies and ADCs are explored as resistance mechanism-agnostic therapeutic approaches with potential promise in the post-osimertinib setting.

Going forward, as treatment strategies become increasingly complex, pragmatic clinical trial designs with broader inclusion criteria reflecting real-world settings will be necessary to yield more clinically applicable evidence. This will allow for incorporation of patient-reported outcomes along with efficacy and safety endpoints to understand their full spectrum of impact. Altogether, these active efforts highlight a future path of adaptive therapies to resistance biology, biomarker-driven strategies, and increasingly individualized and patient-centered approaches.
